# Topical Heparin and Heparinoid-Containing Products as Treatments for Venous Disorders: Compounds, Effects, Clinical Implications, and Recommendations

**DOI:** 10.3390/jcm14061859

**Published:** 2025-03-10

**Authors:** Daniele Bissacco, Chiara Pisani, Gianraffaele Avallone, Ilenia D’Alessio

**Affiliations:** 1 Department of Clinical Sciences and Community Health, University of Milan, Via Francesco Sforza 35, 20122 Milan, Italy; 2 School of Vascular Surgery, University of Milan, Via Francesco Sforza 35, 20122 Milan, Italy; 3 School of Vascular Surgery, University of Naples Federico II, 80138 Naples, Italy; 4 Vascular Surgery Department, Grande Ospedale Metropolitano Niguarda, 20162 Milano, Italy

**Keywords:** chronic venous disease, topical treatment, heparin, glycosaminoglycan

## Abstract

**Background**: Although considerable data are available on oral venoactive drugs, very little information has been published on the types and outcomes of topical treatments for venous disease (VD). This comprehensive review assesses the efficacy and safety of topical heparin and heparinoid-containing products (HCPs) for VD treatment. **Methods**: This narrative review adhered to established methodologies and standards, utilizing the Scale for the Assessment of Narrative Review Articles (SANRA) for quality assessment. A comprehensive literature search was conducted across MEDLINE (PubMed), Scopus, and Web of Science, covering publications from January 1, 1950, to December 1, 2024. Findings were presented in a narrative format, following structured recommendations to ensure clarity and coherence. **Results**: Topical heparin and HCPs provide anticoagulation, enhance microcirculation, and regulate skin permeability, with effects influenced by the concentration and formulation. While they effectively improve skin microcirculation in healthy individuals, research on their intracellular effects is limited. Mucopolysaccharide polysulfate (MPS) in heparinoids offers similar vascular benefits and promotes antithrombotic and anti-inflammatory actions. Moisture and gentle abrasion enhance heparin absorption. Topical heparin and HCPs effectively treat superficial vein thrombosis (SVT) and varicose veins (VVs). Products like Hirudoid significantly alleviate SVT symptoms, including pain and swelling. Clinical trials demonstrate substantial symptom improvement with heparin gel (1000 IU/g). For varicose veins, Xioglican cream stabilizes symptoms and improves quality of life, while other formulations like Essaven Gel and Venoruton enhance microcirculation. **Conclusions**: Managing VD with topical treatments is complicated by the outdated literature and inconsistent methodologies. There is a clear need for systematic research to establish guidelines on the administration, dosage, and frequency of topical treatments. The recommendations in this review aim to provide a foundation for future studies to improve the management of SVT and VVs disease.

## 1. Introduction

Venous disease (VD), which includes a range of both acute and chronic issues such as varicose veins (VVs) and thrombotic venous disorders, is among the most encountered chronic health conditions, contributing significantly to patient morbidity [1,2]. The reported incidence of these venous disorders can vary significantly depending on the specific anatomical region and the classification system used to define the pathology [3]. There are several treatment algorithms for VD care, which vary depending on the type of disease, its grade, and its extent. Among them, heparin plays a crucial role in VD management, not only in varicose veins disease but—more importantly—in thrombotic disorders as well [4].

Moreover, in the perioperative period following an invasive treatment for VD, the use of topical and systemic heparins is essential for managing thromboembolic risk. Topical heparin, applied directly to the treatment site, helps minimize localized clot formation and supports wound healing, particularly in procedures such as sclerotherapy or endovenous laser treatment. Systemic heparin, on the other hand, is crucial for patients undergoing more extensive interventions, such as vein stripping, as it reduces the risk of deep vein thrombosis (DVT) and ensures adequate blood flow in the venous system [5,6].

Heparin is a highly sulfated glycosaminoglycan (GAG) that has been a cornerstone of medical practice for more than 90 years, primarily recognized for its role as an anticoagulant [4]. It is typically administered through parenteral routes, either intravenously or subcutaneously. This compound works by inactivating multiple enzymes involved in the coagulation cascade through its interaction with antithrombin, which is vital for both the prevention and treatment of thrombotic disorders [4,6].

The significance of heparin extends across a wide range of medical procedures and conditions. It is essential in contexts such as kidney dialysis, surgical interventions, and the management of acute events like heart attacks, pulmonary embolism, and strokes [4]. Its versatility and effectiveness in these critical situations underscore its importance in contemporary medicine.

Sodium heparins represent a diverse collection of linear chain anionic mucopolysaccharides recognized for their significant anticoagulant properties. In various countries, their use in phlebology primarily targets conditions such as VVs and superficial vein thrombosis (SVT)/phlebitis and complications arising after surgical procedures [7]. When applied topically, sodium heparin is particularly effective in managing VD, with its predominant action being anti-inflammatory in nature [8]. Topical sodium heparins are also extensively utilized to alleviate symptoms and reduce risks associated with various peripheral vascular disorders.

Furthermore, heparinoid-containing products (HCP) in cream or gel form have demonstrated safety and effectiveness in treating VD and various skin disorders, owing to their anti-inflammatory properties, epidermal permeability barrier, and antimicrobial properties [9,10].

Despite their widespread application, there is a notable scarcity of published research focused on the efficacy and safety of topical heparin treatments.

The main objective of this review is to collate and summarize the existing literature that discusses the application of topical sodium heparins and HCPs in gel/cream form for the treatment of VD. This includes examining patient outcomes in terms of symptom improvement and resolution for any kind of venous disease. Additionally, a secondary goal is to explore the characteristics of effective treatments, including details such as application frequency and dosage, to better understand how these factors influence therapeutic outcomes.

## 2. Materials and Methods

The research methodology and writing standards employed in this narrative review adhered strictly to established protocols and guidelines as detailed in the existing literature. Specifically, we utilized the Scale for the Assessment of Narrative Review Articles (SANRA) [10]. Although SANRA should be used once the review is conducted, we followed its checklist beforehand to better ensure a rigorous quality assessment of the materials included in our study. To gather a comprehensive array of the relevant literature, an extensive search was undertaken across multiple databases, including MEDLINE (PubMed), Scopus, and Web of Science. The temporal scope of our literature search was defined to encompass publications from 1 January 1950, through 1 December 2024. This time frame was carefully selected to capture a broad historical perspective on the subject matter. Only articles published in the English language with an accessible full text were considered for inclusion in our review. To refine our search and enhance the relevance of the retrieved articles, we employed a strategic selection of keywords. These keywords were derived from medical subject headings (MeSH) specific to PubMed and MeSH/EMTREE utilized in Scopus. Free keywords were also employed (e.g., trademark names of topical heparins or HCPs) to better capture relevant articles. The combined keywords included terms such as ‘chronic venous disease’, ‘chronic venous insufficiency’, ‘varicose vein’, ‘leg edema’, ‘deep venous thrombosis’, ‘post-thrombotic syndrome’, ‘venous leg ulcer’, ‘superficial vein thrombosis’, ‘topical heparin’, ‘cream/gel heparin’, ‘mucopolysaccharide cream/gel’, and ‘heparinoids’. This combination was intended to create a robust pool of the literature pertinent to our study. The inclusion criteria for the selected articles were meticulously defined to ensure the relevance and quality of the studies included in the review. Specifically, only studies published in peer-reviewed journals within the established time frame were considered. To maintain a standard of credibility, each study included in the review was required to include a minimum of 10 patients. There were no restrictions placed on the type of study, apart from conference abstracts. Additionally, the studies needed to offer clear and recognizable outcomes.

To enrich our analysis, we also meticulously screened the reference lists of the selected studies for any additional relevant publications that may have been overlooked. Moreover, we reviewed the “Article in Press” sections of various vascular journals, such as *Vascular Medicine*, *Vascular*, *Phlebology*, *Angiology*, *Journal of Vascular Surgery: Venous and Lymphatic Disorders*, *European Journal of Vascular and Endovascular Surgery*, *Annals of Vascular Surgery*, *Journal of Thrombosis and Hemostasis*, and *Thrombosis Research*. This review was conducted with the intent of identifying articles that had not yet been indexed in the primary scientific databases. In addition, we consulted recent guidelines for potential references that could augment our research.

The findings of this review were articulated in a narrative format, following the structured recommendations proposed by Gasparyan et al. [11] and Gregory et al. [12], ensuring clarity and coherence in the presentation of results.

## 3. Results

After the research process, a total of 33 studies were included and presented in a narrative way.

### 3.1. Molecular Properties

According to Cesarone and collaborators [13], heparin applied to the skin exerts three primary effects: (a) anticoagulant action, (b) modulation of microcirculation, and (c) regulation of skin permeability and homeostasis, which enhances the diffusion of other drugs into the skin. These effects are influenced by various factors, including the concentration and formulation of heparin as well as skin characteristics such as temperature, pH, and blood flow.

Although several studies have been published on the clinical effects of topical heparin [14], very few data are available on the intracellular effect. Topical heparin alone has proven quite effective in improving skin microcirculation (capillary venous oxygen saturation SO_2_, blood filling of microvessels, blood flow, and velocity) in healthy subjects [15]. The limited evidence for local heparin administration likely stems from the well-known effects of intravenous heparin. It is generally assumed that similar effects would occur with transdermal application, leading to a lack of focused research on its local use.

**Figure 1:**
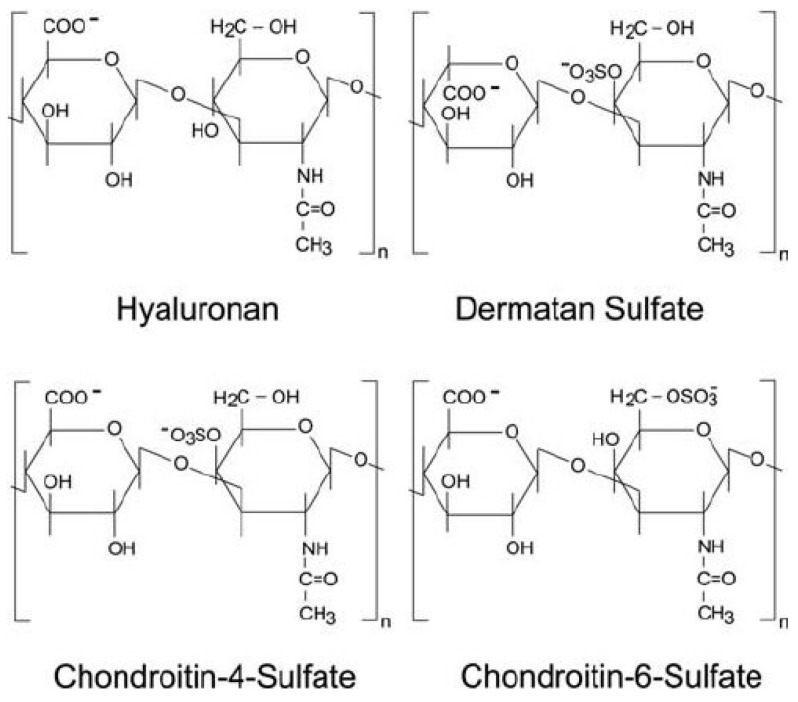
Chemical structures of glycosaminoglycans.

On the other hand, HCPs provide several insights regarding their effects on vessel walls and circulation. HCPs contain mucopolysaccharide polysulfate (MPS), a preparation of GAGs derived from mammalian cartilage that has several structural and functional similarities to heparin. GAGs consist of unbranched polysaccharide chains formed by the repetition of a specific disaccharide unit (Figure 1). As is well known, MPS has numerous critical functions, particularly the regulation of protein activity via cell signaling affecting chemokines, cytokines, growth factors, and adhesion molecules, along with antiproteolytic effects. If administered orally, it modulates several vascular functions in patients with VD, providing antithrombotic, anti-inflammatory, and vascular repair effects [16]. In particular, MPS decreases whole blood viscosity dose-dependently in a manner similar to that of heparin, assuming it can maintain this property even when taken as a cream/gel [17].

Although the outermost layer of skin is typically impermeable, increased moisture levels or gentle abrasion can significantly enhance the absorption of heparin. Stüttgen and collaborators investigated the absorption of heparin and MPS (Hirudoid^®^, STADA Arzneimittel AG, Bad Vilbel, Germany) in human skin samples. They revealed that both substances are absorbed at similar levels in normal skin; however, MPS was absorbed more effectively in keloid skin, which has a thinner epidermis. Additionally, multiple applications of MPS significantly increased its concentration in the deeper layers of the skin. When comparing the two treatments, heparin accumulated more in the epidermis, while MPS levels were higher in the deeper layers when the concentrations in the creams were equivalent [18]. Moreover, an animal model revealed that MPS inhibited thrombus formation in the microvessels of rats, increasing reperfusion of pre-occluded vessels earlier than controls [19]. MPS effects are not only specifically related to the vascular compartment.

Once it penetrates the skin, MPS acts on several intracellular mechanisms, well summarized by a meta-analysis on its effect on skin eczema (Table 1) [20]. Although this table was created to summarize the key findings on the benefits of MPS cream for treating eczema and improving skin health, these treatments may also alleviate VD signs and symptoms. This is because inflammation, which contributes to eczema, is a fundamental mechanism underlying VD-related changes [1,8].

### 3.2. Clinical Effects

The clinical effects of topical heparin and HCPs are numerous. As mentioned, they can be used for skin-related alterations, such as eczema [20], bruising [21], and blunt impact injuries [22]. As per this article’s purpose, the analysis was conducted on the role of topical heparin/HCPs on VD-related diseases, such as SVT and venous incompetence/VVs disease.

#### 3.2.1. Superficial Vein Thrombosis

Topical heparinoids, including products like Hirudoid, are widely used in the treatment of SVT [23], a condition characterized by inflammation and clot formation in superficial veins, often accompanied by pain, swelling, and redness, with a common clinical sign being the presence of a cord-like structure felt upon palpation of the affected vein [24]. These are effective in treating small, localized SVTs, especially in the early stages, while in cases with extensive thrombosis or risk of deep vein involvement, systemic treatment (e.g., anticoagulants) is required.

Hirudoid is clinically recognized for its ability to reduce pain and inflammation. Research has shown its effectiveness in alleviating symptoms/signs of SVT and providing relief in uncomplicated cases [23]. Its anticoagulant and decongestant properties enhance local blood flow, which aids in thrombus reabsorption and minimizes oedema, thereby accelerating recovery. As a topical treatment, Hirudoid has a lower incidence of side effects compared to systemic therapies like low-molecular-weight heparins, with adverse events being rare and typically limited to mild local reactions such as itching or irritation.

As described further below, numerous studies have explored the efficacy and tolerability of various commercially available topical formulations containing heparin and heparinoid substances. These include Lioton^®^ Gel (Menarini, Firenze, Italy), containing heparin 1000 IU/g; Essaven^®^ Gel (Sanofi, Paris, France), formulated with heparin 100 IU/g, aescinate 0.01 g/g, and essential phospholipids 0.01 g/g; LipoHep Forte^®^ (Medicom, Pointe-Claire, QC, Canada) Spray Gel, which features liposomal heparin at 2400 IU/g; Hirudoid^®^ Cream, comprising mucopolysaccharide polysulfate 3 mg/g (classified as a heparinoid); and Movelat^®^ Cream (Thornton & Ross, Slaithwaite, UK), containing organoheparinoid 0.2%, adrenocortical extract 1%, and salicylic acid 2%.

A study involving 32 patients with superficial thrombophlebitis, as primary diagnoses or as complications after sclerotherapy, demonstrated the effectiveness of heparin gel (1000 IU/g) applied over a 4-week period. The treatment led to significant improvements in key symptoms, including induration (hardened tissue), pain, swelling, and overall limb function, when compared to the baseline condition. These findings emphasize the gel’s therapeutic value in managing localized venous inflammation and associated complications [25].

A non-comparative study investigated the use of a heparin cream in 48 patients with superficial thrombophlebitis, primarily chemotherapy-induced (46 cases) or of spontaneous origin (2 cases). The cream was specially prepared by the researchers using commercially available heparin (25,000 IU) mixed with 25 g of an anhydrous lanolin base. Treatment was administered for 7 to 10 days or until the thrombophlebitis resolved [26].

The results showed that most patients (42 out of 48) achieved complete symptom resolution within one week, although statistical comparisons with baseline were not provided. Notably, patients who did not respond to the cream had delayed initiation of treatment, starting therapy more than one week after the onset of symptoms. These findings highlight the importance of timely intervention in managing superficial thrombophlebitis.

A study involving 40 patients with various vascular conditions, including thrombophlebitis and post-phlebitis syndrome, evaluated the effectiveness of twice-daily application of heparin gel (1000 IU/g) compared to a combination heparin/aescinate/phospholipid gel [27]. Both treatments improved symptoms such as oedema, pain, erythema, haematoma, and heaviness, with a higher proportion of patients experiencing symptom relief with heparin gel 1000 IU/g. However, the differences between the two treatments were not statistically significant.

Both therapies also reduced the erythrocyte sedimentation rate (−26.7% with heparin gel 1000 IU/g vs. −28.7% with the combination gel), indicating anti-inflammatory effects. Importantly, neither treatment had any impact on systemic coagulation parameters, supporting their safety for localized use. These findings suggest that heparin gel 1000 IU/g remains a viable option for managing superficial venous conditions [28].

The clinical effectiveness of heparin gel 1000 IU/g was also evaluated in two studies involving patients with venous disorders of the lower limbs. In the first study, 20 patients with recent-onset varicose phlebitis applied the gel three times daily for 15 days, followed by twice daily for an additional 15 days. By days 15 and 30, significant improvements were noted in pain, erythema, and oedema compared to baseline, with no systemic or local adverse events reported [29]. In a comparable study involving 30 patients with superficial thrombophlebitis, varicose phlebitis, or periphlebitis, treatment with heparin gel 1000 IU/g three times daily for 18–25 days (average duration: 20.6 days) resulted in statistically significant improvements (*p* < 0.05 compared to baseline) in spontaneous and induced pain, oedema, erythema, functional limitations, heaviness, paraesthesia, induration, and venous turgor [30].

Comparative studies suggest that Hirudoid provides faster relief from pain and swelling than placebo or non-heparinoid creams. The effectiveness and tolerability of this MPS cream in treating SVT have been confirmed in several placebo-controlled studies. One notable trial evaluated 100 patients with superficial thrombophlebitis caused by infusion therapy. Over a 5-day period, treatment with mucopolysaccharide cream combined with firm bandaging significantly accelerated symptom relief compared to placebo. The mean time to relief of local symptoms was 58 h in the treatment group, versus 126 h in the placebo group (*p* < 0.05) [31]. These findings underscore the cream’s ability to reduce inflammation, improve local circulation, and alleviate discomfort associated with superficial vein inflammation.

A study included 44 patients with vascular conditions such as SVT and post-thrombotic syndrome, who were treated with either heparin gel (1000 IU/g) or MPS cream applied 2–3 times daily for 4–6 weeks [32]. The results indicated that heparin gel (1000 IU/g) achieved a proportionally greater improvement compared to mucopolysaccharide cream. This included significant reductions in spontaneous and induced pain, limb oedema, and feelings of heaviness. Evaluations by both investigators and patients showed an improvement rate of 99% with heparin gel compared to 63% with mucopolysaccharide cream. Importantly, no adverse skin reactions were noted in either treatment group, and neither product affected systemic coagulation parameters, supporting their safety profiles. However, it is worth noting that no statistical analysis was conducted in this study, which limits the strength of the conclusions drawn.

According to the studies mentioned above, treatment with Hirudoid and other topical heparinoids has proven to be effective, safe, and well-tolerated in the management of uncomplicated SVT. In more severe cases, however, these treatments can be used in combination with systemic therapies to ensure a more comprehensive and effective therapeutic approach.

#### 3.2.2. Venous Incompetence/Varicose Vein Disease

Topical treatment for varicose veins and/or saphenous incompetence utilizes heparins/HCPs to reduce some of the VD-related symptoms. In a prospective observational study, Muratori et al. evaluated the efficacy and safety of Xioglican^®^, (Neopharmed Gentili S.p.A., Milan, Italy) cream in patients with CEAP class C2-C3 VD [33]. Xioglican cream is an HCP containing galactosaminoglycan polysulfate and hyaluronic acid with strong hydrophilic, moisturizing, and soothing properties. Topical treatment with Xioglican cream was associated with stabilizing or reducing some VD-related skin signs and symptoms (i.e., oedema, itching, pain, and swelling), although there was no change in CEAP classification after the treatment. An improvement in subjective measures of VD, including Venous Clinical Severity Score (VCSS) and patients’ Quality of Life (QoL), was also observed, suggesting that Xioglican was associated with stabilization of the disease. Cesarone and collaborators, in two different works, have studied the effects of the topical application of a gel including aescin, essential phospholipids (EPL), and heparin (Essaven Gel^®^) on transcutaneous PO_2_ in venous insufficiency and venous hypertension microangiopathy (VHM) due to varicose veins. As before, a placebo-controlled randomized study showed that topical application with hand massage of EG improves local microcirculation on skin with VHM, with a decrease in skin flux and CO_2_ and an increased O_2_ [28]. These findings suggest that continuous treatment may be beneficial and prevent complications such as ulcerations. A second study—based on PO_2_ evaluations obtained by measuring transcutaneous PO_2_ in the perimalleolar region—has shown that the topical application of gel on skin with venous microangiopathy results in an improvement in microcirculation and skin perfusion with a significant improvement in signs/symptoms [34]. These findings are related to the anti-oedema and venoactive properties of aescin and the effects of EPL in reducing platelet aggregation and improving blood viscosity and microcirculation.

A prospective comparative study by Belcaro and coworkers studied the effects of topical application of another compound called O-(β-hydroxyethyl)-rutosides (HR, Venoruton^®^, STADA Arzneimittel AG, Bad Vilbel, Germany) [35]. HR demonstrated effectiveness in improving microcirculation and various venous parameters. Endothelial cells showed positive responses to HR, and the QoL for patients with chronic venous insufficiency improved with HR compared to other treatments. After 8 weeks of treatment, symptomatic improvement was linked to decreases in capillary filtration, foot oedema, and swelling, along with enhanced skin flux at rest, assessed by measuring transcutaneous PO_2_ and PCO_2_.

Several comparative studies reviewed by Vecchio and Frisinghelli examined the effects of heparin gel (1000 IU/g) in patients with various vascular disorders, including chronic venous disease and varicose ulcers [24]. The findings indicated that patients treated with heparin gel experienced a more significant improvement in signs and symptoms compared to those receiving MPS cream or a gel containing heparin, aescinate, and phospholipids. Notably, the symptoms most commonly reported by patients—such as feelings of heaviness, cutaneous dyscrasias, erythema, and pain from haematomas—were markedly alleviated in the heparin gel group.

## 4. Discussion

Gathering robust evidence from the existing literature on topical treatments for venous diseases poses a significant challenge. This complexity arises from a variety of factors.

Firstly, much of the available literature is outdated and limited in scope, which undermines the ability to draw comprehensive conclusions. Additionally, many studies lack rigorous methodologies, including randomized controlled trials, which are essential for ensuring the reliability and validity of the findings. Moreover, there is a wide array of compounds that have been tested, making it difficult to compare results across different studies. This variability complicates the process of establishing standard criteria for grading and assessing the signs and symptoms associated with venous diseases, such as VVs and SVT. Consequently, the combination of these factors creates a landscape where evidence synthesis is not only challenging but also often inconclusive, highlighting the need for more systematic research in this area.

Despite these challenges, some considerations can be made regarding the use of local heparin and HCPs. Evidence suggests that these treatments have demonstrated a sort of effectiveness, although not always tied to a specific clinical outcome. Instead, their benefits are more broadly related to the relief of signs and symptoms associated with venous diseases. In light of this review, it can be recommended that the use of local heparin or HCPs is particularly appropriate for patients suffering from SVT. Furthermore, these treatments may also be beneficial for selected symptomatic patients dealing with VVs. This recommendation is based on the overall positive impact these therapies have shown in alleviating discomfort and improving the QoL for individuals affected by these conditions.

The current understanding of the optimal design, methodology, and frequency of topical treatments for VD remains somewhat underdeveloped and inadequately discussed. Specifically, there is a lack of clarity regarding how these treatments should be administered, when they should be initiated, and how often they should be applied for maximum effectiveness. It is generally accepted that treatment should commence as soon as possible after the onset of signs and/or symptoms to achieve the best outcomes. Research has demonstrated that gently massaging the affected area until the cream or gel is fully absorbed significantly enhances the absorption of the active ingredients. Therefore, this technique is recommended for patients using topical treatments. While some dosage regimens have been proposed, a common recommendation for patients with SVT is to apply the topical treatment one to two times per day until there is complete remission of the signs and symptoms. This approach aims to facilitate faster recovery and symptom relief. In contrast, the literature does not provide robust recommendations for the use of topical treatments in patients with VVs. Consequently, more comprehensive research is necessary to establish clear guidelines for dosage and frequency in this patient population.

Although topical treatments have demonstrated effectiveness in managing venous diseases, it is crucial that they are utilized in conjunction with other therapeutic modalities. This comprehensive approach should include a combination of additional topical treatments, such as elastic stockings, as well as non-topical therapies, including venoactive drugs.

Furthermore, the application of invasive interventions may be necessary, depending on the severity, extent, and potential complications associated with the specific venous condition being treated.

Indeed, topical treatments alone should not be recommended if SVT or VVs are diagnosed. Table 2 summarizes the recommendations based on the authors’ considerations regarding the management of VD with topical treatments, according to the selected literature.

## 5. Conclusions

Managing VD with topical treatments is complicated by outdated literature and inconsistent methodologies. While local heparin and HCPs show potential for relieving symptoms in conditions such as SVT and, to a lesser extent, VVs, robust evidence for their use remains limited. Prompt initiation of treatment and techniques to enhance absorption are essential. However, topical treatments should not be used in isolation; a comprehensive approach that includes additional therapies and invasive interventions is necessary for optimal patient outcomes.

There is a clear need for more systematic research to establish definitive guidelines on the administration, dosage, and frequency of topical treatments. The recommendations presented in this review aim to provide a foundation for future studies, ultimately enhancing the management of venous diseases.

## Figures and Tables

**Table 1:** Summarizing the effects of mucopolysaccharide polysulfuric acid ester (MPS) cream on patients with eczema [20].

Mechanism	Description	Effects
Restoration of Skin Barriers	MPS cream reduces transepidermal water loss (TEWL) and enhances the expression of epidermal mRNA related to lipid production (HMGCoA, FAS, SPT1).	Increased levels of key skin proteins: filaggrin, involucrin, and loricrin. Mitigates TCS-induced TEWL.
Anti-inflammatory Effects	MPS cream lowers total IgE and thymic stromal lymphopoietin (TSLP) levels in serum. It reduces infiltration of mast cells and CD3+ T cells in lesions.	Decreased expression of pro-inflammatory cytokines (IL-4, IL-6, IL-13, IL-22). Suppresses IL-1ß production.
Enhancement of Adjunct Treatments	Supports the anti-inflammatory effects of topical corticosteroids (TCS) or tacrolimus ointment (TAC-O).	Enhances overall treatment efficacy when used alongside TCS or TAC-O.
Antimicrobial Activity	MPS cream upregulates mouse beta-defensin 3 (mBD3) expression in the epidermis, an antimicrobial peptide against Gram-negative bacteria and Candida.	Suggests potential for improved skin infection management.

**Table 2:** Key recommendations and considerations for effectively managing venous diseases while addressing the limitations in the current literature. HCP, Heparinoid-Containing Product; VVs, Varicose Veins; SVT, Superficial Venous Disease.

Recommendation	Details
Utilize Topical Treatments Concurrently	Combine local heparin or HCP with other therapies for comprehensive management.
Incorporate Additional Topical Treatments	Use adjunctive treatments such as elastic stockings alongside topical agents to enhance therapeutic effects.
Consider Non-Topical Therapies	Include venoactive drugs in the treatment plan to address venous disease holistically.
Initiate Treatment Promptly	Begin topical treatment as soon as symptoms arise to maximize effectiveness and optimize patient outcomes.
Enhance Absorption Through Massage	Gently massage the affected area until the cream or gel is fully absorbed to improve the absorption of active ingredients.
Follow Recommended Dosage for SVT	Apply topical treatment one to two times per day until complete remission of signs and symptoms in patients with SVT.
Limited Guidance for VVs	Acknowledge that the current literature lacks robust recommendations for dosage and frequency of topical treatments in VVs; further research is needed.
Assess Severity for Invasive Interventions	Evaluate the need for invasive procedures based on the severity, extent, and potential complications of the venous condition.
Avoid Sole Reliance on Topical Treatments	Do not recommend topical treatments as the sole therapy for diagnosed SVT or VVs; a multimodal approach is essential.
